# Shoulder-Pad Sign in a Case of Amyloidosis Associated with Myeloma

**DOI:** 10.4274/tjh.galenos.2021.2021.0630

**Published:** 2021-08-25

**Authors:** Ceren Uzunoğlu, Tayfur Toptaş, Yıldız İpek, Fatma Arıkan, Fergün Yılmaz, Tülin Tuğlular

**Affiliations:** 1 Marmara University Training and Research Hospital, Clinic of Internal Disease, İstanbul, Turkey; 2 Marmara University Training and Research Hospital, Clinic of Hematology, İstanbul, Turkey

**Keywords:** Shoulder pad, Multiple myeloma, Kappa light chain, AL amyloidosis

## To the Editor,

**Figure 1:**
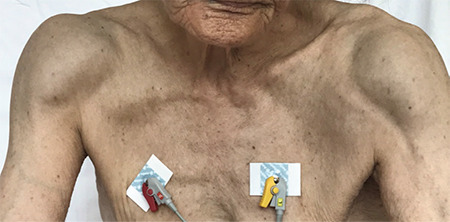
Joint examination revealed swelling and tenderness of the bilateral anterior parts of the shoulders, elbows, wrists, knees, and ankles.

A 78-year-old female was admitted with the complaints of multiple joint swellings that had progressed over 8 months, involving the shoulders, elbows, wrists, knees, and ankles. On physical examination, she had generalized edema. The tongue was enlarged. Joint examination revealed swelling and tenderness of the bilateral anterior parts of the shoulders, elbows, wrists, knees, and ankles (Figure 1).

Her complete blood count and calcium levels were as follows: leukocytes, 5100/µL; hemoglobin, 5.2 g/dL; hematocrit, 16%; platelets, 313,000/µL; serum calcium, 11.9 mg/dL. Serum total protein and albumin levels were 4.6 g/dL and 3.0 g/dL, respectively. Serum free light chain kappa, lambda, and kappa/lambda ratio were 6792 mg/L, 7.31 mg/L, and 929, respectively. Serum immunofixation electrophoresis was consistent with the kappa monoclonal band. Proteinuria (5 g) was identified upon 24-h urine analysis.

**Figure 2:**
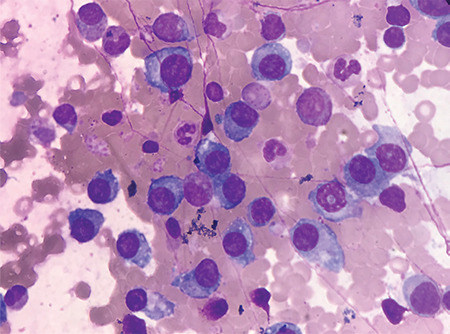
Bone marrow biopsy showed plasma cells infiltrating 86% of bone marrow.

**Figure 3:**
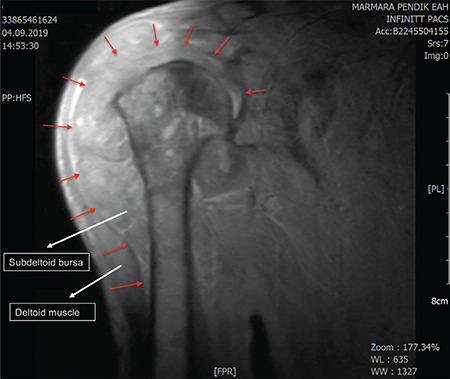
Significant thickening of the subdeltoid bursa was evident upon shoulder magnetic resonance imaging.

Bone marrow biopsy showed plasma cells infiltrating 86% of bone marrow (Figure 2). No lytic bone lesions were detected on positron emission tomography-computed tomography, but there were joint involvements compatible with inflammatory processes. Significant thickening of the subdeltoid bursa was evident upon shoulder magnetic resonance imaging (Figure 3). Bone marrow biopsy with Congo red staining revealed a green birefringent color representing amyloid deposition. A diagnosis of systemic amyloidosis associated with multiple myeloma was made.

“Shoulder-pad sign” is the prominent appearance of the bilateral anterior deltoid area. It is rare but suggestive for amyloid light-chain amyloidosis. It is mostly associated with the kappa light chain, which has a predilection for amyloid deposition in periarticular tissues [1].

In amyloidosis, joint or soft tissue involvement rarely occurs [2]. It was reported that 3.7% of 191 patients with systemic amyloidosis had amyloid arthropathy and the shoulders were the most commonly affected joints [3]. Due to symmetrical joint involvement with pain, swelling, and limitation of movement, rheumatologic diseases might be considered in the differential diagnosis.
